# An update on the molecular genetics toolbox for staphylococci

**DOI:** 10.1099/mic.0.061705-0

**Published:** 2013-03

**Authors:** Marcel Prax, Chia Y. Lee, Ralph Bertram

**Affiliations:** 1 Department of Microbial Genetics, Interfaculty Institute of Microbiology and Infection Medicine Tübingen (IMIT), Faculty of Science, University of Tübingen, Waldhäuser Str. 70/8, 72076 Tübingen, Germany; 2 Department of Microbiology and Immunology, University of Arkansas for Medical Sciences, 4301 W. Markham Street, Slot 511, Little Rock, AR 72205, USA

## Abstract

Staphylococci are Gram-positive spherical bacteria of enormous clinical and biotechnological relevance. *Staphylococcus aureus* has been extensively studied as a model pathogen. A plethora of methods and molecular tools has been developed for genetic modification of at least ten different staphylococcal species to date. Here we review recent developments of various genetic tools and molecular methods for staphylococcal research, which include reporter systems and vectors for controllable gene expression, gene inactivation, gene essentiality testing, chromosomal integration and transposon delivery. It is furthermore illustrated how mutant strain construction by homologous or site-specific recombination benefits from sophisticated counterselection methods. The underlying genetic components have been shown to operate in wild-type staphylococci or modified chassis strains. Finally, possible future developments in the field of applied *Staphylococcus* genetics are highlighted.

## Introduction

Among the Gram-positive bacteria, the staphylococci comprise more than 70 species and subspecies and form a distinct monophyletic group within the Firmicutes (Ghebremedhin *et al.*, 2008; Takahashi *et al.*, 1999; Pantůček *et al*., 2013) (Leibniz Institute DSMZ-German Collection of Microorganisms and Cell Cultures, January 2013). Staphylococci are spherical bacteria with a diameter of 0.5–1.8 µm and have a low G+C DNA content of approximately 33–40 mol%. They are generally facultatively anaerobic and catalase-positive, among many other distinctive characteristics (Götz *et al.*, 2006). Undoubtedly the most prominent representative of this genus is *Staphylococcus aureus*, which resides in the nares of approximately 30 % of the world’s human population (DeLeo *et al.*, 2010). Usually asymptomatic, *S. aureus* is capable of causing superficial to serious infections of almost all tissues, particularly in immunocompromised individuals (Lowy, 1998). Strains resistant to most clinically applicable antibiotics, commonly referred to as meticillin resistant *S. aureus* (MRSA), pose an especially serious health risk (Levy & Marshall, 2004). The enormous wealth of different *S. aureus* strains and lineages is reflected by a large number of frequently used laboratory strains, such as Newman, COL, USA300, UAMS-1 and the NCTC8325 (RN1)-derived strains SH1000, 8325-4, SA113, RN4220 or the HG series (Herbert *et al.*, 2010). As original or genetically modified clinical isolates, they differ markedly with regard to pleiotropic transcriptional regulators, activity of the *agr* quorum sensing system, physiological fitness, availability and activity of virulence factors or genetic amenability and robustness. This underscores the need to particularly verify findings on gene regulation phenomena in different genetic backgrounds before drawing conclusions universally valid in *S. aureus. Staphylococcus epidermidis*, another opportunistic pathogen, is usually part of the normal skin flora of healthy individuals but is also associated with high numbers of catheter or other foreign body related bloodstream infections (Otto, 2009). Of note, not all staphylococci are pathogenic and some have been exploited in food industry and biotechnology. For example, *S. carnosus*, *S. xylosus* and *S. equorum* contribute to cheese ripening or meat fermentation, or serve as a chassis for protein production in biotechnology (Corbiere Morot-Bizot *et al.*, 2007; Götz, 1990; Place *et al.*, 2003). The need to combat pathogenic species and to optimize *Staphylococcus* strains as production vehicles (e.g. to produce higher yields or to streamline genomes for enhanced stability) requires further understanding of these bacteria and efficient methods for genetic manipulations. The use of molecular genetic tools in staphylococci has been described previously (McNamara, 2008; Novick, 1991). Here we provide an updated overview on well-established and new molecular methods on how gene expression in staphylococci can be monitored or modulated and summarize approaches to inactivate genes or to engineer staphylococcal genomes.

## Plasmids and transformation

Plasmids are essential for most bacterial genetics tools. The magnitude of vectors used for Staphylococci are derived from the naturally occurring plasmids pC194, pE194, pT181 and pUB110 or related elements, which are copied by a rolling circle mechanism, or pI258 and pSK1, which employ the theta-mode of replication. Due to the repletion of different plasmids used for cloning in staphylococci, we will merely focus on a limited number of key constructs applicable as *Staphylococcus*/*E. coli* shuttle vectors. In early studies, the ori (origin of replication) of small rolling circle plasmids, such as pUB110, was harnessed for hybrid plasmid vectors (Brückner *et al.*, 1984; Brückner, 1992). Subsequently, vectors derived from plasmid pSK1 (Firth *et al.*, 2000), which may confer higher segregational and structural stability, have been reported (Grkovic *et al.*, 2003). A series of versatile cassette-based pCN vectors have been established (Charpentier *et al.*, 2004) whereby fragments for plasmid replication (based upon wild-type or modified pT181-ori or pI258-ori), antibiotic selection and maintenance in *E. coli* can be exchanged in a modular fashion. These plasmids have different features including copy numbers, temperature sensitivity in replication, ability to integrate into a specific site of the chromosome and Cd^2+^-inducible gene expression. Vectors primarily applied for inducible gene expression or allelic replacement are described in later sections of this article; for more comprehensive overviews on other well-established *Staphylococcus* vectors, the reader is referred to reviews by McNamara (2008) and Novick (1989).

Unlike a number of *Bacillus* or *Streptococcus* species, laboratory grown *S. aureus* usually appears to be incapable of taking up foreign DNA despite harbouring homologues of competence genes (Morikawa *et al.*, 2003). However, as reported only recently, expression of the *sigH* gene renders *S. aureus* cells competent (Morikawa *et al.*, 2012) and it will be interesting to see whether this finding can be exploited for applied *Staphylococcus* genetics. As of now, *S. aureus*, *S. epidermidis*, *S. staphylolyticus* and *S. carnosus* are usually transformed by using various electroporation protocols or occasionally protoplast transformation (Augustin & Götz, 1990; Götz & Schumacher, 1987; Löfblom *et al.*, 2007). However, restriction–modification (RM) systems of most *S. aureus* strains act as an efficient barrier against foreign DNA (Monk & Foster, 2012; Monk *et al.*, 2012; Waldron & Lindsay, 2006; Xu *et al.*, 2011). For transformation of foreign DNA into *S. aureus*, strain RN4220, which is deficient in type I restriction HsdR but retains the modification function, has been extensively used as an initial recipient before transferring DNA to other strains (Kreiswirth *et al.*, 1983). However, many strains accept DNA isolated from RN4220 at an extremely low frequency, if at all. The type IV RM factor SauUSI, which is highly conserved in *S. aureus* and cleaves a sequence motif containing a methylated cytosine residue, has later been found to be a major barrier for DNA uptake in *S. aureus*. Monk *et al.* (2012) therefore constructed an *E. coli* strain, DC10B, that lacks the cytosine methylation system *dcm.* They observed successful transformation of plasmids isolated from DC10B without using RN4220 as the intermediate host. The transformation rate is further improved using DNA isolated from strain SA30B, in which the *S. aureus* type I RM genes *hsdMS* were cloned into DC10B (Monk & Foster, 2012).

## Reporter genes

Reporters are invaluable tools for profiling the spatio-temporal activity of genes or proteins which themselves do not show an apparent or easy to assay phenotype. There is no shortage on reporter genes to choose from, depending on the specific purpose. Reporter genes used in staphylococci include *xylE* of *Pseudomonas putida*, *lip* of *S. hyicus*, *cat* of plasmid pC194, *blaZ* of pI258, and *lacZ* of *E. coli* (Table 1). Although some of these reporters are still in use, proteins or enzymes conferring fluorescence or luminescence are gaining popularity due to their ease of monitoring. Initially, the *Photinus pyralis* firefly luciferase (*luc*), which requires luciferin as a substrate and the bacterial *luxAB*-encoded luciferase, which uses substrates such as *n*-decyl aldehydes, had been used as reporters in *S. aureus* (Corbisier *et al.*, 1993; Meighen, 1993; Murray *et al.*, 2001; Steidler *et al.*, 1996). However, the inclusion of *luxCDE* genes encoding a fatty acid reductase complex for the intracellular production of aldehydes as substrate eliminates the exogenous substrate for the LuxAB luciferase reporter (Meighen, 1991). To enhance the production of fatty acid aldehydes, the *luxCDE* genes were placed under the control of the constitutive *ami* promoter of *Streptococcus pneumoniae* (Mesak *et al.*, 2009). This improved reporter system was used to screen for anti-staphylococcal compounds (Mesak *et al.*, 2010). The *lux* system is also suitable for tracing an *S. aureus* infection in a mouse model by measuring the *in vivo* bioluminescence of a respective strain. Optimization of the ribosome-binding site of each of the five *lux* genes derived from *P. luminescens* resulted in an improved bioluminescent *S. aureus* strain detectable in a mouse model (Francis *et al.*, 2000).

**Table 1.** Reporters

Gene(s)	Function	Substrate	Origin	Remarks*	Reference†
*xylE*	Catechol 2,3-dioxygenase	Catechol	*P. putida*	Generally requires cell disruption	Sheehan *et al.* (1992)
*lip*	Lipase	Tributyrin or tween	*S. hyicus*	–	Wieland *et al.* (1995)
*blaZ*	β-lactamase	Nitrocefin	*Staphylococcus* plasmid pI258	–	Wang *et al.* (1987)
*cat*	Chloramphenicol acetyltransferase	Chloramphenicol	*Staphylococcus* plasmid pUB112	Requires cell disruption	Otto *et al.* (1998)
*lacZ*	β-Galactosidase	X-Gal	*E. coli*	–	Ohlsen *et al.* (1997)
*bgaB*	β-Galactosidase	X-Gal	*B. stearothermophilus*	Used for qualitative assays only	Arnaud *et al.* (2004)
*luc*	Luciferase	Luciferin	*Photinus pyralis*	–	Steidler *et al.* (1996)
*luxAB/luxABCDE*	Luciferase	n-decyl aldehydes/–	*Vibrio harveyi* or *Photorhabdus luminescens*	No exogenous substrate required, when *luxABCDE* genes used	Corbisier *et al.* (1993), Mesak *et al.* (2009)
*gfp* (variants)	Auto-fluorescent protein	–	*Aequorea victoria* and synthetic variants	Numerous derivatives with altered properties, substrate independent	Cheung *et al.* (1998)

* All listed reporters allow for a direct or coupled photometric readout in a mostly quantitative fashion (exceptions marked).

† Reference in regard to first published application in *Staphylococcus*.

It should be noted that the application spectrum of these enzyme-based systems is confined due to low spatial resolution capacity. The use of cofactor- and substrate-independent fluorescent proteins circumvents this limitation. Particularly, the green-fluorescent protein (GFP) is a popular and approved reporter in many bacteria to study not only promoter activity (Malone *et al.*, 2009), which could be optionally coupled to flow cytometer aided high-throughput analysis (Southward & Surette, 2002), but also protein localization. The triple mutant GFP_UV_ was exploited to assess the transcriptional profile of the global regulatory *sar* locus in the vegetation tissues of rabbits with experimental infective endocarditis (Cheung *et al.*, 1998). In a later study, the red-shifted variant GFP3 was expressed in *S. aureus* and other Gram-positive species (Qazi *et al.*, 2001b). Due to the inducible P*_xylA_* promoter and an optimized ribosome-binding site, a strong fluorescence signal could be induced in staphylococci. Live imaging via fluorescence microscopy facilitates tracing the movement of GFP-fused single proteins and their localization within a bacterial cell (Pereira *et al.*, 2010). On a larger scale, intravital imaging allows fluorescent protein tagged bacteria to be subcutaneously localized, as demonstrated in infected mice (Liese *et al.*, 2012).

Recently, several new fluorescent reporters have been employed in staphylococci which differ in emission wavelengths [mCherry, YFP, GFP, CFP (Malone *et al.*, 2009; Pereira *et al.*, 2010)] and excitation maxima [GFP_UVR_ (Kahl *et al.*, 2000)]. Some of them are codon-optimized or photoactivatable (Paprotka *et al.*, 2010; Sastalla *et al.*, 2009) or exhibit different folding characteristics (Pédelacq *et al.*, 2006; Yu & Götz, 2012). Autofluorescent reporters are often very stable, as reflected by a half-life of ~7 h in case of Gfpmut3.1 in *S. epidermidis* (Andersen *et al.*, 1998; Franke *et al.*, 2007), which precludes their use in time-resolved expression studies. GFPmut3 derivatives with short peptide tags intended to stimulate proteolytic degradation were found to glow very weakly, apparently caused by low protein levels (Franke *et al.*, 2007). It is conceivable that low stability fluorescent protein variants used in other Gram-positive bacteria might be functional in staphylococci as well. For example, Qazi *et al.* (2001b) developed a GFP3 variant with a half-life of approximately 54 min in *Listeria monocytogenes* and destabilized eYFP proteins were exploited in *Corynebacterium glutamicum* (Hentschel *et al.*, 2012). The use of a dual system, combining the *gfp* and the *lux* systems, revealed that a bioluminescence signal was only detectable in growing staphylococci with a high metabolism, reflecting the expression of a gene in real-time, whereas GFP fluorescence increased at a later time point and remained constant for more than 10 h (Qazi *et al.*, 2001a). These two systems in combination facilitate the monitoring of both living and dead cells of the same sample (Qazi *et al.*, 2004).

## Inducible gene expression

A cornucopia of induction systems for bacteria is available to produce heterologous proteins, to decipher gene function relationships or to reveal gene dosage effects (Terpe, 2006) and there is also no shortage of options for staphylococci (Table 2). The β-lactamase-inducible promoter, the arsenite-inducible promoter and the cadmium-inducible promoter, all from pI258, have been explored for inducible expression in *S. aureus* (Vandenesch *et al.*, 1991; Corbisier *et al.*, 1993; Charpentier *et al.*, 2004). A xylose-inducible promoter of *S. xylosus* was pioneered by the laboratory of Friedrich Götz to be used in gene regulation studies. Advantage was taken by the fact that *S. xylosus* possesses genes for xylose utilization unlike many staphylococci which can only internalize xylose (Kloos *et al.*, 1991; Sizemore *et al.*, 1991). A series of vectors, known as pCX, pKX and pTX, were developed that mostly differ in resistance markers, plasmid backbone, copy number and regulation capacities, as has been demonstrated in *S. aureus* (Hussain *et al.*, 2001; Krismer, 1999; Peschel *et al.*, 1996; Wieland *et al.*, 1995). This system can be induced by 0.5 % xylose, but alternatively can also be repressed by 0.5 % glucose due to catabolite repression (Hueck *et al.*, 1994; Sizemore *et al.*, 1992). A similar induction system operative in the staphylococcal vector pPSHG3 relies on the transcriptional repressor GalR of the *S. carnosus* galactose utilization gene cluster *galRKET*. High level production of heterologous cytoplasmic or secreted proteins upon galactose addition was achieved particularly in an *S. carnosus* Δ*galRKET* background, in which the cytoplasmic inducer is not metabolized (B. Krismer, unpublished results). Another carbohydrate controllable system that has been used in staphylococci is the P*spac* hybrid promoter that is repressed by the *lac* operon regulator LacI and induced by IPTG (Yansura & Henner, 1984).

**Table 2.** Inducible gene expression systems

Target promoter	Regulator	Effector(s)	Origins of genetic components	Remarks	Reference*
P-*bla*	BlaI	Carboxyphenylbenzoyl-aminopenicillanic acid	*Staphylococcus* plasmids pI524 and pI258	–	Vandenesch *et al.* (1991)
Pcad	CadC	Cd^2+^	*Staphylococcus* plasmid pI258	–	Corbisier *et al.* (1993)
Pars	ArsR	Arsenite	*Staphylococcus* plasmid pI258	–	Corbisier *et al.* (1993)
P*_xylA_*	XylR	Xylose, glucose	*S. xylosus*	Induced by xylose, repressed by glucose	Kloos *et al.* (1991)
P*_galKET_*	GalR	Galactose	*S. carnosus*	Improved efficacy in *S. carnosus* Δ*galRKET*	B. Krismer (unpublished data)
P*spac*	LacI	IPTG	*E. coli*, hybrid promoter	–	Halfmann *et al.* (1993)
P_xyl/tet_	TetR, revTetR	Anhydrotetracycline (ATc), TetR-inducing-peptide (Tip)	Gram-negative Tc resistance determinants, hybrid or synthetic (rev)TetR variants, hybrid promoters	TetR: induction with ATc or Tip (‘Tet-ON’), revTetR: corepression with ATc (‘Tet-OFF′)	Gauger *et al.* (2012); Ji *et al.* (1999)
Pro3	C1 repressor	Thermal shift, 31 °C to 42 °C	Bacteriophage P1, synthetic promoters	Repressed at 31 °C, induced at 42 °C	Schofield *et al.* (2003)
P_T7_	T7 RNAP	–†	Bacteriophage T7	–	D’Elia *et al.* (2006)

* Reference in regard to first published application in *Staphylococcus*.

† Transcriptional induction of the T7 RNA polymerase (RNAP) encoding gene by IPTG.

A different common gene induction system that has gained popularity is based upon the artificial P_xyl/tet_ hybrid promoter (Geissendörfer & Hillen, 1990), which can be induced at a much higher level, for example, than the P*spac* promoter (Zhang *et al.*, 2000). P_xyl/tet_ is accrued from the *Bacillus subtilis* P_xylA_ promoter by placing a *tet* operator (*tetO*) between the −35 and the −10 element. *tetO* is the cognate sequence of the tetracycline (Tc) repressor TetR, which is involved in controlling Tc efflux determinants in Gram-negative bacteria (Grkovic *et al.*, 2002). Anhydro-Tc (ATc), a potent effector of TetR, is frequently applied at a final concentration of 0.4 µM (corresponding to approximately 200 ng ml^−1^) to ensure complete induction, whereas lower inducer amounts (such as 100 ng ml^−1^) are sufficient for many applications and reduce the risk of growth inhibition. An important property of the P_xyl/tet_ system to be considered is that it can be regulated under *in vivo* conditions in *S. aureus*-infected mice by Tc-supplemented drinking water (Ji *et al.*, 1999). The *tet*-regulatory system is also useful for inducible antisense RNA expression and has been shown to be functional in single copy level when integrated at a defined or random site of the chromosome (Gründling & Schneewind, 2007a, b). Cloning of the *tet*-regulatory architecture into a pC194-derived vector yielded the popular *tet*-control shuttle vector pALC2073 (Bateman *et al.*, 2001) to inducibly express *sigB* and many more target genes. This plasmid’s leakiness in the non-induced state was reduced in two recent studies. Corrigan & Foster (2009) enhanced the promoter driving *tetR* in plasmid pRMC2 (which resulted in elevated repressor amounts), and subsequently Helle *et al.* (2011) added a second *tetO* sequence to P_xyl/tet_ yielding pRAB11. Additionally, mutating P_xyl/tet_ at between one and four distinct positions of the −10 and/or −35 sites leads to lower expression levels upon induction (Helle *et al.*, 2011; Xu *et al.*, 2010) to better reflect strengths of native *S. aureus* promoters. With variable regulation capacities, plasmids pRMC2, pRAB11 and the related pCG248 (Corrigan & Foster, 2009; Helle *et al.*, 2011) represent second generation *tet*-regulation constructs for staphylococci. Also, the reverse TetR (revTetR) system was applied in *S. aureus* (Stary *et al.*, 2010). In this system, two mutations within the TetR change ATc into a corepressor. Thus, interaction of ATc with revTetR results in a rapid shut-down of the promoter (Kamionka *et al.*, 2004; Scholz *et al.*, 2004). *tet*-regulation has found broad application to identify essential *S. aureus* genes and to validate candidate drug targets in the pharmaceutical industry (Huber *et al.*, 2009; Ji *et al.*, 2001). Recently, a protein monitoring system exploiting *tet*-components was adapted for *S. aureus* (Gauger *et al.*, 2012). Therein, a gene of interest is translationally fused to a sequence encoding a short and specific peptide stretch, which is capable of acting as an alternative inducer of TetR (Klotzsche *et al.*, 2005). Increasing abundance of the deduced tagged protein therefore leads to a fluorescent signal via de-repression of a P_xyl/tet_ controlled fluorescent reporter gene. An overview on the use of *tet*-regulation in bacteria is provided by Bertram & Hillen (2008) and Bertram (2010).

Physical, compound-independent induction of transcription in *S. aureus* has been achieved by employing the bacteriophage P1 temperature-sensitive C1 repressor. It was demonstrated that *lacZ* driven by artificial promoters equipped with two C1 binding sites could be controlled between a temperature range of 31 (repressed) and 42 °C (induced) (Schofield *et al.*, 2003). Another induction system, which abandons the use of a bacterial RNA polymerase-dependent promoter upstream of a gene of interest and instead relies on bacteriophage T7 RNA polymerase-dependent transcription, has been employed in *S. aureus* (D’Elia *et al.*, 2006). A sequence containing the T7 polymerase-encoding gene downstream of a P*spac* promoter adjacent to a constitutively expressed *lacI* was planted into the *geh* locus in the chromosome for controlling T7-driven target genes. Compared to its bacterial multi-subunit counterpart, T7 RNA polymerase is monomeric, has a considerably reduced molecular mass (100 kDa) and confers faster transcription (Cheetham & Steitz, 2000). A post-transcriptional mode of gene activity control in bacteria is operated by riboswitches in which RNA molecules undergo conformational changes upon small-molecule binding that could, for example, conditionally shield ribosome-binding sites (Wittmann & Suess, 2012). In *B. subtilis* and *Streptococcus pyogenes*, heterologous riboswitches responsive to theophylline have been successfully employed for gene regulation when placed in 5′ untranslated regions of transcripts (Topp *et al.*, 2010). A number of riboswitches have been found in *S. aureus* (Felden *et al.*, 2011) and although untapped so far, this proteinogenic regulator free architecture renders riboswitches a possible option for artificial gene regulation in staphylococci in the future.

## Systems for allelic replacement

Chromosomal inactivation is the principal approach to decipher a bacterial gene’s function. The majority of methods for inactivating a chromosomal gene exploit the cells’ homologous recombination RecBCD machinery. The canonical process of allelic replacement starts with cloning genomic fragments flanking the gene of interest into a deletion vector. Insertion of a marker gene between the flanking sequences facilitates later identification of desired mutants, which can be cumbersome, particularly, if allelic replacement systems of low efficiency are used. The size of the flanking regions should be 0.4 kb or more; empirically, longer fragments may increase recombination (McNamara, 2008). After transformation of the target strain, the recombinant plasmid is intended to undergo single or consecutive double crossover recombination events, resulting in DNA replacement in the chromosome. A pioneering study was conducted in the laboratory of Timothy Foster when *hla* in *S. aureus* strain 8325-4 was chromosomally inactivated by insertion of an erythromycin cassette of plasmid pE194 (O’Reilly *et al.*, 1986). Since the first success of allelic exchange in staphylococci, several deletion vectors functioning via the same basic principle have been described. Among them, the temperature-sensitive *S. aureus/E. coli* shuttle vector pBT2 (Brückner, 1997) has been frequently applied for gene replacement in *S. aureus*, *S. xylosus*, *S. carnosus* and *S. epidermidis* as well as in the human pathogen *S. lugdunensis* and the lantibiotic producer *S. gallinarum*, as reported recently (Krismer *et al.*, 2012; Marlinghaus *et al.*, 2012). The pBT2 vector backbone, which carries a *cat* gene and the temperature sensitive pE194ts replicon, permits stable inheritance in staphylococci at 30 °C. Chromosomal integration of pBT2-derived knockout plasmids is expedited by a thermal upshift to temperatures of about 42 °C, which are non-permissive for pBT2. Akin to the strategy employed by O’Reilly *et al.* (1986), desired strains devoid of the gene of interest are then screened for chloramphenicol sensitivity and selected for resistance to another antibiotic. Frequently, a gene conferring resistance against erythromycin, tetracycline or kanamycin is chosen as a marker. The thermal shift, typically between 42 and 30 °C, is a key step to achieving successful allelic exchange for temperature sensitive vectors. Interestingly, shifting between 42 and 25 °C twice was found to dramatically increase the rate of plasmid loss after integration (Kato & Sugai, 2011). However, the underlying mechanism is unknown. Allelic exchange methods relying on incubation steps at temperatures non-physiological to staphylococci are prone to second site mutations. It is hence recommended to transfer mutated loci to another strain. This can be accomplished by serogroup B bacteriophages (Stewart *et al.*, 1985), with ϕ11, 52a, 80α and L54a being preferred vectors.

In order to facilitate mutant identification after allelic replacement, the pMAD system was developed (Arnaud *et al.*, 2004). pMAD is composed of fragments from plasmids pE194ts and pBR322 and carries the *B. stearothermophilus bgaB* gene encoding β-galactosidase under a constitutive promoter. After a temperature-dependent allelic exchange procedure, colony colours on X-Gal plates report on the status of pMAD: blue indicates that the plasmid is still in the episomal state, light blue represents colonies with chromosomal integration of the entire vector sequence (single crossover) and colourless suggests successful replacement (double crossover). The pMAD system thus allows one to screen presumptive mutants for further confirmation thereby increasing the rate of success in mutant construction.

The use of counterselectable markers can be useful to accelerate the identification of desired knockout candidates and a number of such markers are available for staphylococci (Table 3). Systems for substrate-independent counterselection exploit antisense RNA fragments directed against an essential gene or the activity of an endonuclease. Bae & Schneewind (2006) developed the plasmid pKOR1 that efficiently reduced the background of wild-type colonies grown on solid media after an allelic replacement procedure. At the heart of the system is a *tet*-inducible antisense *secY* fragment located on the backbone of a vector with a pE194ts ori. Encoding part of the Sec translocon, *secY* is essential and thus its antisense RNA leads to the loss of *S. aureus* viability. pKOR1 includes two *attP* sites of the Gateway system (Invitrogen/Life Technologies) for ligase-independent cloning of flanking regions in *E. coli* and the *ccdB* gene encoding a toxic protein to eliminate cells bearing insert-free plasmids. Allelic exchange in *S. aureus* is achieved by temporarily confined cultivation in the presence of antibiotic selection under non-permissive conditions for pKOR1 (43 °C) to select for plasmid integration. Enrichment of desired knockout candidates is achieved by spreading cells onto ATc-containing agar, whereupon transcription of antisense *secY* RNA is induced. The same counterselection device acts in plasmid pIMAY (Monk *et al.*, 2012), which is equipped with a pVW01ts ori for replication up to 30 °C, a multiple cloning site and a strong promoter derived from *Lactococcus lactis* to drive the *cat* gene for selection. The latter feature presumably aids in selection of correct single-copy integrands. A third plasmid making use of antisense *secY* counterselection is pBASE6, which is derived from fusing a fragment of pKOR1 with parts of pBT2 (Geiger *et al.*, 2012). pBASE6 appears to exhibit enhanced stability during cloning in *E. coli* and has successfully been used for allelic replacement in *S. aureus*, *S. epidermidis* and *S. lugdunensis* (B. Krismer, unpublished results). Both pIMAY and pBASE6 include dedicated restriction sites for conventional cloning and are considerably smaller than pKOR1, which has advances for transformation and stability.

**Table 3.** Markers for counterselection

Factor or protein	Function, mode of action	Substrate→product	Origin	Remarks	Reference*
Antisense *secY* RNA	Downregulation of essential *secY*	*secY* mRNA→dsRNA (prone to degradation)	*S. aureus*	Induced by ATc, targeting of essential *secY* gene	Bae & Schneewind (2006)
I-SceI	DNA endonuclease	DNA→site-specifically cleaved DNA	*Saccharomyces cerevisiae*	Sequence specific cleavage of DNA	Pagels *et al.* (2010)
SacB	Levansucrase	Sucrose→levan	*B. amyloliquefaciens*	Secretion of variant *sacB*[*BamP*]W29 is impaired	D’Elia *et al.* (2006)
GdmP	Pregallidermin protease	Pregallidermin→gallidermin	*S. gallinarum*	Validated for *S. carnosus*	Krismer *et al.* (2012)
PyrE and PyrF	Orotate phosphoribosyltransferase (PyrE) and orotidine 5-phosphate decarboxylase (PyrF)	5-FOA→5-fluoro-UMP	*B. subtilis*	–	Redder & Linder (2012)

* Reference in regard to first published application in *Staphylococcus*.

Another strategy employed for counterselection to facilitate mutant generation is the homing endonuclease I-SceI (Pósfai *et al.*, 1999). To use this system in *S. aureus*, a 30 bp fragment containing the large I-SceI recognition site was cloned into the pBT2-derived construct pJM930 and the endonuclease was constitutively expressed from a second compatible plasmid termed pJM928 (Pagels *et al.*, 2010). Upon a temperature shift and subsequent introduction of pJM928 by transduction, pJM930 is cleaved by I-SceI either in the episomal state or after single crossover chromosomal integration. With no I-SceI enzyme recognition site, the *S. aureus* chromosome remains otherwise unaffected. The double stranded DNA break promotes the second homologous recombination step required for the resolution of the plasmid cointegrate to generate the desired mutant by a statistical 50 % chance. Several alternative counterselection systems have been described that facilitate allelic exchange in staphylococci requiring either carbohydrates, nucleotide analogue or lantibiotic precursors as substrates. An early example is represented by plasmid pSAKO (D’Elia *et al.*, 2006), which uses the *sacB*[*BamP*]W29 gene, encoding a variant of the *B. amyloliquefaciens* levansucrase SacB for counterselection in *Bacillus* (Bramucci & Nagarajan, 1996). SacB catalyses the polysaccharide levan in the presence of sucrose, which is toxic to many bacteria for reasons not entirely understood. Wild-type *sacB* is a widely applied marker for counterselection in several bacteria, but for *S. aureus* the use of a variant with a defective secretion signal sequence appears to be critical. A system for allelic replacement in *S. carnosus* (Krismer *et al.*, 2012) employs the lantibiotic precursor pregallidermin, naturally produced by *S. gallinarum* (Kellner *et al.*, 1988) and the cognate protease GdmP which converts pregallidermin to the toxic form by removal of a leader peptide. By exploiting a pBT2 derivative bearing *gdmP*, plasmid-free mutants were achieved in pregallidermin spiked medium. As a result, only cells cured from the episomal or chromosomally integrated plasmid are expected to form colonies on respective agar plates. When tested by PCR in the course of *srtA* (sortase) mutant construction, all of the 16 tested colonies grown on pregallidermin-containing plates were correct. A technique validated in *S. aureus* has been developed which is based on the degradation of the pyrimidine-analogue 5-fluoroorotic acid (5-FOA) (Redder & Linder, 2012). The genes *pyrE* and *pyrF*, whose encoded proteins convert 5-FOA to the toxic product 5-fluoro-UMP, were incorporated into these deletion vectors. Addition of 5-FOA to *S. aureus* harbouring plasmid pRLY2 or derivatives therefore allows counterselection. More than a dozen of these allelic replacement vectors have been constructed which exhibit different antibiotic markers and origins of replication. In the same study, recombinant strains facilitating the deletion process were also developed by moving the *repC* gene of the pT181 replication machinery of the plasmid vector into the chromosome of the restriction-deficient strain RN4220. The resulting strain allowed a *repC*-deleted vector to replicate and to be appropriately modified by methylation. Upon subsequent transformation into a wild-type strain, the plasmid became a suicide vector but its DNA would not be restricted. Such a replication-dependent host/vector system is therefore useful for mutant construction similar to other suicide vectors. It should be noted here that such strategy was employed earlier by Xia *et al.* (1999) for constructing non-replicative suicide vectors for testing gene essentiality (see below).

A mechanistically distinct method for targeted disruption of *S. aureus* genes is based on the commercially available TargeTron Gene Knockout System (Sigma) that has been used in various bacteria. In this system, *Lactococcus lactis* Ll.LtrB mobile group II introns are tailored to insert into a gene of interest at specific sites selected based on a computer algorithm. Targeted gene disruption occurs by an RNA–protein complex that mediates reverse splicing of the intron RNA into the desired locus, followed by a reverse transcription step. The system has been adapted for use in *S. aureus* by incorporating Cd^2+^-dependent induction of the targetron (Yao *et al.*, 2006). Gene disruption by TargeTron was reported to occur even in the uninduced state and most likely depends on the recognition ability of the retargeted intron, which may differ between genes of interest.

## Gene essentiality testing

Essential genes are important targets for antibiotic drug development. These genes, by definition, are involved in various critical cellular functions for bacterial survival that cannot be inactivated. Thus, one way to determine whether a gene is essential is to determine whether a loss-of-function mutation of the gene can be achieved. One strategy to study *S. aureus* essential genes is to use a temperature-sensitive shuttle vector carrying the 5′ end of the target gene in which the start codon was replaced with a stop codon and select for single-crossover integration at 42 °C in the presence of selective pressure (Yamachika *et al.*, 2012). As a result, the plasmid integrants bear a truncated copy of the target gene and a full-length inactive variant devoid of the start codon. Integrations at essential genes are therefore lethal and respective strains cannot be obtained. This strategy is similar to the method developed earlier that employs a replication gene-dependent suicide vector carrying an internal fragment of a gene of interest (Xia *et al.*, 1999). A relatively new method to test gene essentiality based on the targetron system discussed above has been recently reported (Yao *et al.*, 2006; Zoraghi *et al.*, 2010). In this method, the insertion of the intron is carried out in both the sense and antisense orientations. Several parameters can be used to assess whether a target gene is essential. Firstly, because insertion of a targetron in the sense, but not in the antisense, orientation allows the intron RNA to splice out from the transcribed mRNA (thereby restoring the original mRNA sequence) mutant strains could only be obtained when the insertion is in the sense orientation if the target gene is essential. On the other hand, if the target gene is non-essential, mutants could be obtained regardless of the insertion orientation of the intron. Secondly, since LtrA is required for RNA splicing, curing of the delivery plasmid encoding LtrA would also be lethal for insertions in an essential gene. Lastly, since the splicing reaction is temperature-sensitive (Yao *et al.*, 2006), strains with targetron insertions in an essential gene would not survive at 43 °C. These systems described above, however, suffer from an inherent problem associated with inactivating essential genes as the respective deletion strains cannot be cultured and thus merely provide indirect proofs. This limitation is circumvented by a number of systems that employ other strategies.

A more direct approach to demonstrate gene essentiality is to place the gene of interest under the control of an inducible promoter in the chromosome or in a low-copy plasmid and assess the essentiality based on survival in the presence or absence of the inducer (Fan *et al.*, 2001; Jana *et al.*, 2000; Liew *et al.*, 2011; Zheng *et al.*, 2005). The IPTG-inducible P*spac* promoter has been favoured in these systems. However, overproduction of the LacI repressor from a multi-copy plasmid is required for proper repression of the P*spac* promoter. Although straightforward, this procedure requires time-consuming chromosomal manipulations. By contrast, antisense technology that acts post-transcriptionally leaves a native gene of interest unaffected in the chromosome. The principle is simple: an antisense RNA of the target gene is (usually episomally) expressed from an inducible promoter with conditional growth of the resulting strain as readout. The ability to grow in the presence but not in the absence of the inducer indicates that the gene of interest is essential. All methods described here use inducible promoters (see above). Thus, the quality of the inducible promoters, such as leakiness and inducibility, will affect the testing results.

Global approaches have been employed to comprehensively identify essential genes in *S. aureus*. More than 150 essential genes have been identified by antisense technology (Forsyth *et al.*, 2002; Ji *et al.*, 2001). A random transposon mutagenesis strategy was used to identify 351 putative essential genes devoid of transposon insertions from a large mutant library (Chaudhuri *et al.*, 2009). However, considerable discrepancy exists among the lists of essential genes, which likely stems from different methods or conditions used in the experimental procedures. Thus, a gene categorized as ‘essential’ may not be truly essential especially if it is inherently expressed at a level that is outside the range of a controllable promoter. Verification is always recommended preferably with two complementary methods. Another caution for essential gene testing is the potential polarity effect of operonic genes that are common in bacteria.

## Site-specific recombination systems

Site-specific recombination (SSR) is required for a large number of processes in bacteria, particularly involving mobile genetic elements. In contrast to homologous recombination, specialized recombinases operate to break and rejoin covalent bonds of nucleic acids (Grindley *et al.*, 2006). Natural staphylococcal plasmids also bear SSR genes that can facilitate formation and resolution of cointegrates or plasmid multimers (Gennaro *et al.*, 1987). SSR systems have been exploited in applied bacterial genetics for targeted chromosomal integration of DNA fragments, genome rearrangements or genomic deletions, to name just a few examples. A number of systems have also been harnessed for genome modification in staphylococci (Table 4). One of the examples is the single-copy integration system developed two decades ago, which is still in use today (Lee *et al.*, 1991). The vectors are based on the SSR of lysogenic bacteriophage L54a that integrates into the *attB* attachment site on the chromosome located within the *geh* gene (Lee & Iandolo, 1986a, b). These vectors, which lack an ori for replicating in *S. aureus*, carry the L54a phage attachment *attP* site for integration catalysed by integrase expressed either from the vector in the case of pCL55 or from an auxiliary plasmid pYL119Δ19 in the case of pCL83 or pCL84 (Lee *et al.*, 1991). The original system was further improved by including a second attachment site derived from bacteriophage ϕ11 to generate pLL29 and pLL39 (Luong & Lee, 2007). This facilitates alternative integration into another chromosomal locus containing the ϕ11 *attB* site (which inserts at a hypothetical gene) if the L54a attachment site is already occupied or if lipase-positive strains are required e.g. for virulence studies. Based on the backbone of pCL83/84, the SSR of bacteriophage ϕ13, which inserts the *S. aureus hlb* gene (encoding β-haemolysin), has also been used to construct a new integration vector (Mainiero *et al.*, 2010). Another pCL84 derivative is pKASBAR, which has a smaller plasmid size and improved multiple-cloning site to facilitate cloning (G. McVicker and S. Foster, personal communication). Also, Benton *et al*. (2004) constructed pCL84-derived components to identify virulence genes of *S. aureus*. The usefulness of these integration vectors for studies that require chromosomal equivalent gene dosage is, however, accompanied by the disadvantage of the inactivation of bacterial genes that harbour these integration sites, which might have undesired effects. To solve this problem, the DNA components required for L54a integration were redesigned to produce a new and orthogonal *attB*/*attP* pair by altering the core sequence at five identical positions (Lei *et al.*, 2012). The newly engineered *attB2* site was artificially planted into an intergenic region of the *S. aureus* genome devoid of measurable transcriptional activity. The improved vector pLL102 with the newly engineered *attP2* was shown to integrate specifically at the cognate *attB2* site.

**Table 4.** Site-specific recombination and transposition-based systems

Recombinase	Cognate nucleic acid sequence(s)	Origin	Remarks	Reference*
L54a integrase	L54a *attP* (>27 bp), *attB* (228–235 bp) or variants thereof	Bacteriophage L54a and synthetic sequences	Integration only	Lee *et al.* (1991), Lei *et al.* (2012)
ϕ11 integrase	ϕ11 *attP* (~25 bp), *attB* (<394 bp)	Bacteriophage ϕ11	Integration only	Luong & Lee (2007)
ϕ13 integrase	ϕ13 *attP* (~26 bp), *attB* (<252 bp)	Bacteriophage ϕ13	Integration only	Mainiero *et al.* (2010)
SaPI1 integrase	*att_S_* (~17 bp)	*S. aureus*	Integration only	Charpentier *et al.* (2004)
Cre recombinase	*loxP* or variants thereof (34 bp)	Bacteriophage P1 and synthetic sequences	Integration, inversion or excision; used in *Staphylococcus* for excision only	Leibig *et al.* (2008)
γδ resolvase	*res* (~114 bp)	Transposon γδ	Used in *S. aureus* for excision only	Lowe *et al.* (1998)
Himar1 transposase	*mariner* terminal inverted repeat (~27 bp)	*mariner*, *himar1* transposon group	–	Bae *et al.* (2004)
Tn*5* transposase	Mosaic element (19 bp)	Tn*5* and hybrid transposase binding sites	Applicable as transposomes	Blake & O'Neill (2013)
Mu transposase	Mu R-end (50 bp)	Bacteriophage Mu	Applicable as transposomes	Pajunen *et al.* (2005)

* Reference in regard to first published application in *Staphylococcus*.

The SSR function of the 15 kb *S. aureus* pathogenicity island 1 (SaPI1) was also exploited to develop single-copy vectors. A 906 bp segment, containing the SaPI1 attachment site *att_S_* was cloned into the temperature sensitive plasmids of the pCN series (Charpentier *et al.*, 2004). This gave rise to constructs pRN7145 and pRN7146, which insert into the chromosome with the help of plasmid pRN7023 expressing SaPI1 integrase.

In addition to the SSR systems derived from staphylococcal phages or pathogenicity islands, the Cre recombinase of coliphage P1, which uses two 34 bp *lox* DNA sites as substrates, has also been applied for use in staphylococci. The Cre-*lox* system is operative with just one enzyme catalysing integration or excision in a co-factor-independent fashion. If each *lox* site is present on two circular DNA molecules, Cre catalyses fusion, resembling plasmid integration into a circular chromosome. However, in the case of two intramolecular *lox* sites, their relative orientation dictates the Cre-dependent outcome: the *lox* flanked DNA is inverted if the sites converge or diverge, whereas the segment is excised if the *lox* sites have the same direction. Due to its versatility, context independence and ease of use, the Cre-*lox* system is widely applied in eukaryotes and prokaryotes (Sauer, 2002). Cre recombinase was used to remove *lox*-flanked resistance markers from genetically modified *S. aureus* and *S. carnosus* strains (Leibig *et al.*, 2008). Direct repeats of *lox* sequences were attached to marker genes *aphAIII* (kanamycin selection) or *ermB* (erythromycin selection). Efficient excision of the antibiotic markers was achieved by expression of *cre*, driven by the *B. anthracis* P*_pagA_* promoter from pRAB1 after 30 °C incubation. A subsequent shift to 42 °C resulted in efficient pRAB1 curing to eventually yield a strain with just one reminiscent *lox* site in the genome. Another heterologous SSR system, resolvase of transposon γδ, has been exploited as readout for promoter activity in *S. aureus* (Lowe *et al.*, 1998). In this study, DNA fragments of a chromosomal *S. aureus* library were first ligated upstream of the promoterless *tnpR* gene encoding the resolvase of transposon γδ, which recognizes and cleaves 122 bp *res* sites. A *res*-*aphAIII*-*res* kanamycin resistance cassette was integrated into the chromosome of *S. aureus*, so that fragments containing active promoters would induce resolvase-dependent *aphAIII* excision. This system was used to identify genes active during the course of infection in which bacterial cells, after 4 days of a renal mouse infection, were collected and checked for kanamycin sensitivity as an indication of active promoters. The system was observed to work reliably and efficiently, as no excision occurred with promoterless *tnpR*, whereas *tnpR* under control of a constitutive promoter led to 100 % resolution.

## Transposition-based systems for mutant generation

Linking a phenotype to a hitherto unknown gene is facilitated by forward genetics, i.e. unspecific mutagenesis and subsequent analysis of a mutant’s outcome. Transposons and bacteriophages are promising tools in this regard, particularly those with random insertion properties for unbiased mutagenesis. Generally, both the complexity of a mutant collection and the randomized distribution of affected loci over the entire genome can contribute to identifying a desired phenotype. An extensive *S. aureus* mutant library was constructed and screened for virulence genes by Bae *et al.* (2004) who had developed the element *bursa aurealis*. It employs features of the high activity variant *himar1* that can be traced back to a group of *mariner* transposons (Lampe *et al.*, 1996). The original *bursa aurealis* protocol for mutagenesis of *S. aureus* Newman requires two plasmids: pBursa contains an *ermB* resistance cassette together with a promoterless *gfp*, flanked by *mariner* terminal inverted repeats, a *cat* gene and a temperature sensitive ori. Integration of the element into the genome downstream of a promoter thus yields a green fluorescence signal. Transposition requires the Himar1 transposase carried by the second temperature-sensitive plasmid pFA545. In this study, randomness of integration was compared to the well-established Tn*917* mutagenesis system, which had, among other applications, been used for signature-tagged mutagenesis of staphylococci (Grueter *et al.*, 1991; Mei *et al.*, 1997). An observation of target sites suggested relatively unbiased insertion of *bursa aurealis* compared to Tn*917*, the latter of which exhibited two target hot-spots in the *S. aureus* genome. Whereas more than 10 000 clones with defined insertion sites were generated in this study [the ΦNΞ (Phoenix) library covers ~67 % of all predicted ORFs (Bae *et al.*, 2004)], a similar approach for saturating *bursa aurealis* mutagenesis of an USA300 derived MRSA strain has recently been undertaken to yield the ‘Nebraska Tn Mutant Library’, which is publicly available for either the entire set of mutants or selected strains thereof (Fey *et al*., 2013). In the course of the study, a number of vectors for allelic replacement, including selectable and fluorescent markers, were also constructed (Bose *et al*., 2013). Since the temperature upshift steps during *bursa aurealis* mutagenesis may cause undesired second-site mutations (see also above for allelic exchange methods), phage transduction of the mutation of interest to a target strain is recommended. Technical specifications and further instructions on the use of this *bursa aurealis* system are detailed by Bae *et al.* (2008). A single plasmid construct for *himar1* mutagenesis, pBTn, has been constructed that bears the respective transposase gene under a xylose-inducible promoter and an erythromycin resistance cassette flanked by *himar1* terminal inverted repeats (Li *et al.*, 2009).

Like *mariner*, transposon Tn*5* is mobilized by a conservative cut-and-paste mechanism (Reznikoff *et al.*, 1999). A hyperactive triple mutant of the Tn*5* transposase together with improved cognate DNA binding sites, so called mosaic elements (ME) (Reznikoff, 2003), was used to construct the vector pTN11. It encompasses both the transposase gene and a ME flanked kanamycin resistance marker and was used to identify *S. aureus* SH1000 mutants impaired in biofilm formation (Lauderdale *et al.*, 2009). An *in vitro* approach developed for *E. coli* mutagenesis with Tn*5*-derived integrative elements uses purified hyperactive Tn*5* transposase that forms ternary complexes with linear DNA fragments terminated by ME elements (Goryshin *et al.*, 2000). These so-called transposomes are stable enough for electroporation into bacterial cells. Here, intracellular Mg^2+^ ions activate the transposase proteins to randomly insert the delivered DNA into the chromosome. Such integrative elements equipped with a resistance marker and an outward facing P_xyl/tet_ promoter (Bertram *et al.*, 2005) were successfully applied to generate more than 20 000 mutant strains of *S. aureus* SH1000 which were entirely screened for enhanced susceptibility to antistaphylococcal agents (Blake & O'Neill, 2013). Besides Tn*5*, a number of other transposases are also utilizable for *in vitro* transposition (Hayes, 2003). Components of the bacteriophage Mu were exploited to subject *S. aureus* strains ATCC 29213 and S30 to transposome mutagenesis (Pajunen *et al.*, 2005). *In vitro*-assembled mini-Mu elements composed of a selective marker and bracketed by the 50 bp Mu R-ends were electroporated into bacterial cells with subsequent Mg^2+^-dependent activation like the Tn*5* transposomes. The efficiency of this method for *S. aureus* mutagenesis was quoted with up to 2×10^4^ antibiotic-resistant colonies per microgram of DNA complexed in transposomes. Since transposome mutagenesis does not require transposase to be genetically encoded in the target organism at any time, resulting insertion mutants exhibit unparalleled genetic stability. Critical factors for transposome mutagenesis include careful analysis and adjustment of optimal molar ratios between transposase protein and cognate DNA, thorough desalting and use of appropriate concentrations of protein–DNA complexes to be subjected to transposome-optimized electroporation.

## Conclusions and future perspectives

A remarkable wealth of new molecular genetic tools for staphylococci has been developed. Due to its clinical relevance, the vast majority of respective studies focus on *S. aureus* and hence most of the tools outlined in the present article are primarily functional in, but not necessarily confined to, this species. Genetic manipulations in other staphylococci are frequently impeded by the lack of efficient transformation protocols, native genetic elements interfering with available vector systems or other limiting factors, including cell envelope composition or DNA methylation patterns. Indeed, only a fraction of *Staphylococcus* species has been reported to be amenable to genetic manipulations. According to the literature and personal information, these currently include (in alphabetical order): *S. aureus*, *S. capitis*, *S. carnosus*, *S. epidermidis*, *S. gallinarum*, *S. intermedius*, *S. lugdunensis*, *S. saprophyticus*, *S. schleiferi* and *S. xylosus*. Of note, the efficiency of molecular tools and methods varies drastically among strains and species, with *S. capitis* or *S. gallinarum* as just two particularly recalcitrant examples. Optimizing transformation protocols may be one critical step to overcome these limitations. In terms of allelic replacement, recombineering approaches using considerably shorter flanking sequences for homologous recombination may facilitate mutant construction. Bacteriophage-derived recombinase systems such as RecET or λ red which exploit phage proteins for single strand DNA binding and annealing have revolutionized mutant strain construction in *E. coli* (Datsenko & Wanner, 2000; Zhang *et al.*, 1998), whereby sequence stretches for homologous recombination are so short that they can be attached by PCR primers. Related approaches have already been successful in Gram-positive bacteria as well (van Kessel & Hatfull, 2008; van Pijkeren & Britton, 2012) and the adaptation of this principle would provide a new powerful tool for applied *Staphylococcus* genetics.
